# Leading Causes of Death in Nonmetropolitan and Metropolitan Areas— United States, 1999–2014

**DOI:** 10.15585/mmwr.ss6601a1

**Published:** 2017-01-13

**Authors:** Ernest Moy, Macarena C. Garcia, Brigham Bastian, Lauren M. Rossen, Deborah D. Ingram, Mark Faul, Greta M. Massetti, Cheryll C. Thomas, Yuling Hong, Paula W. Yoon, Michael F. Iademarco

**Affiliations:** 1 National Center for Health Statistics, CDC; 2 Center for Surveillance, Epidemiology, and Laboratory Services, CDC; 3 National Center for Injury Prevention and Control, CDC; 4 National Center for Chronic Disease Prevention and Health Promotion, CDC

## Abstract

**Problem/Condition:**

Higher rates of death in nonmetropolitan areas (often referred to as rural areas) compared with metropolitan areas have been described but not systematically assessed.

**Period Covered:**

1999–2014

**Description of System:**

Mortality data for U.S. residents from the National Vital Statistics System were used to calculate age-adjusted death rates and potentially excess deaths for nonmetropolitan and metropolitan areas for the five leading causes of death. Age-adjusted death rates included all ages and were adjusted to the 2000 U.S. standard population by the direct method. Potentially excess deaths are defined as deaths among persons aged <80 years that exceed the numbers that would be expected if the death rates of states with the lowest rates (i.e., benchmark states) occurred across all states. (Benchmark states were the three states with the lowest rates for each cause during 2008–2010.) Potentially excess deaths were calculated separately for nonmetropolitan and metropolitan areas. Data are presented for the United States and the 10 U.S. Department of Health and Human Services public health regions.

**Results:**

Across the United States, nonmetropolitan areas experienced higher age-adjusted death rates than metropolitan areas. The percentages of potentially excess deaths among persons aged <80 years from the five leading causes were higher in nonmetropolitan areas than in metropolitan areas. For example, approximately half of deaths from unintentional injury and chronic lower respiratory disease in nonmetropolitan areas were potentially excess deaths, compared with 39.2% and 30.9%, respectively, in metropolitan areas. Potentially excess deaths also differed among and within public health regions; within regions, nonmetropolitan areas tended to have higher percentages of potentially excess deaths than metropolitan areas.

**Interpretation:**

Compared with metropolitan areas, nonmetropolitan areas have higher age-adjusted death rates and greater percentages of potentially excess deaths from the five leading causes of death, nationally and across public health regions.

**Public Health Action:**

Routine tracking of potentially excess deaths in nonmetropolitan areas might help public health departments identify emerging health problems, monitor known problems, and focus interventions to reduce preventable deaths in these areas.

## Introduction

In 2014, approximately 15% of the U.S. population (46 million persons) lived in nonmetropolitan counties (*1*). Nonmetropolitan and metropolitan communities differ in their demographic, environmental, economic, and social characteristics, which influence the magnitude and types of health problems they have. Nonmetropolitan areas have higher rates of cigarette smoking, hypertension, obesity, and physical inactivity during leisure time (*2*–*4*). More residents of nonmetropolitan areas lived in poverty compared with residents of metropolitan areas in 2014 (18.1% and 15.1%, respectively) (*1*). Availability of resources for preventive services and access to health care also differ among certain localities. Residents of nonmetropolitan areas are more likely to report less access to health care and lower quality of health care (*5*). Metropolitan areas generally have a greater density and diversity of health care providers than nonmetropolitan areas (*2*,*3*).

Both in nonmetropolitan and metropolitan areas, the five leading causes of death in the United States during 1999–2014 were heart disease, cancer, unintentional injury, chronic lower respiratory disease, and stroke (*6*) which together accounted for 1,622,304 deaths (approximately 62% of all deaths) in 2014 (*6*). Four of the five leading causes of death were chronic diseases, two of which (heart disease and cancer) accounted for approximately 46% of all deaths each year (*6*). Potentially excess deaths (also described as potentially preventable deaths) (*7*,*8*) are defined as deaths among persons aged <80 years in excess of the number that would be expected if the death rates for each cause were equivalent across all states to those that occurred among the three states with the lowest rates. Not all potentially excess deaths can be prevented; some areas might have characteristics that predispose residents to higher rates of death, such as long travel distances to specialty and emergency care or exposures to specific environmental hazards. However, many potentially excess deaths might represent deaths that could be prevented through improved public health programs that support healthier behaviors and neighborhoods or better access to health care services.

To examine differences for the five leading causes of death in nonmetropolitan and metropolitan areas in the United States, CDC analyzed mortality data from the National Vital Statistics System. This report presents trends in age-adjusted death rates among persons of all ages during 1999–2014 and the number of potentially excess deaths among persons aged <80 years during 2010–2014. These findings can be used by public health officials and practitioners to identify nonmetropolitan areas with the highest rates of potentially excess deaths and to focus on specific regions that would benefit from programs to reduce mortality from the five leading causes of death.

## Methods

Nonmetropolitan and metropolitan areas were identified using the Office of Management and Budget’s 2013 county-based classification scheme (*9*). Although the terms rural and nonmetropolitan often are used interchangeably, as are the terms urban and metropolitan, other definitions of rural exist (*10*). To avoid confusion, the terms nonmetropolitan and metropolitan are used in this report. The term locality is used to refer both to nonmetropolitan and metropolitan areas.

Mortality data for U.S. residents from the National Vital Statistics System were analyzed. Deaths were categorized as nonmetropolitan or metropolitan on the basis of the county of residence of the decedent. Analyses were restricted to deaths with an underlying cause of death among the five leading causes based on the *International Classification of Diseases, 10th Revision* (ICD-10): heart disease (I00-I09, I11, I13, and I20–I51), cancer (C00–C97), unintentional injury (V01–X59 and Y85–Y86), chronic lower respiratory disease (J40–J47), and stroke (I60–I69). The analysis of trends in age-adjusted death rates during 1999–2014 included all ages; death rates were adjusted to the 2000 U.S. standard population by the direct method. The analysis of potentially excess deaths during 2010–2014 was restricted to persons aged <80 years at the time of death; the age restriction is consistent with the average life expectancy for the total U.S. population, which was approximately 79 years in 2010 (*7*).

**FIGURE 1:**
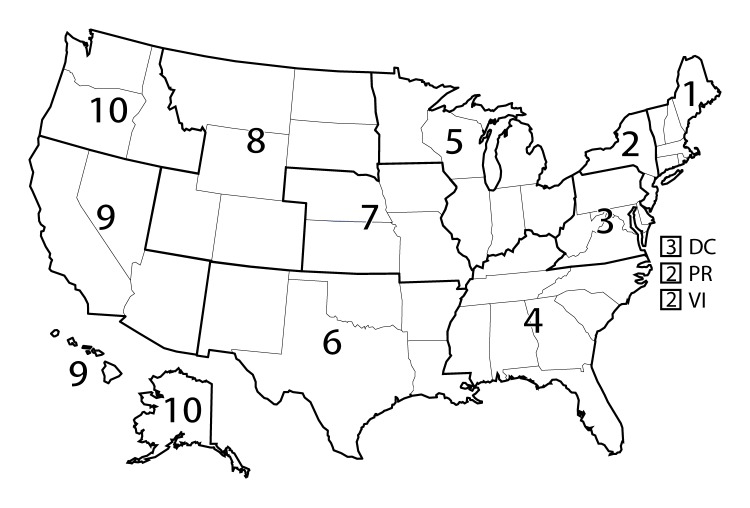
U.S. Department of Health and Human Services public health regions* * *1* = Connecticut, Maine, Massachusetts, New Hampshire, Rhode Island, and Vermont; *2* = New Jersey, New York, Puerto Rico, and the U.S. Virgin Islands (Mortality data for residents of U.S. territories were excluded.); *3* = Delaware, District of Columbia, Maryland, Pennsylvania, Virginia, and West Virginia; *4* = Alabama, Florida, Georgia, Kentucky, Mississippi, North Carolina, South Carolina, and Tennessee; *5* = Illinois, Indiana, Michigan, Minnesota, Ohio, and Wisconsin; *6* = Arkansas, Louisiana, New Mexico, Oklahoma, and Texas; *7* = Iowa, Kansas, Missouri, and Nebraska; *8* = Colorado, Montana, North Dakota, South Dakota, Utah, and Wyoming; *9* = Arizona, California, Hawaii, and Nevada; *10* = Alaska, Idaho, Oregon, and Washington.

Potentially excess deaths were defined as deaths among persons aged <80 years in excess of the number that would be expected if the age-specific death rates of the three states with the lowest rates (i.e., benchmark states) occurred across all states. Numbers of potentially excess deaths for each of the five leading causes of death were calculated using procedures previously published by CDC (*7*). For each age group and cause, the death rates of the three states with the lowest rates during 2008–2010 (benchmark states) were averaged to produce benchmark rates (https://stacks.cdc.gov/view/cdc/42342). Because the goal of this analysis was to calculate total numbers of potentially excess deaths in nonmetropolitan and metropolitan areas, the same benchmarks were applied both to nonmetropolitan and metropolitan areas, and benchmarks were not adjusted for other characteristics that might affect death rates (e.g., race, ethnicity, and socioeconomic status). Estimates of expected deaths for each specific age group, cause of death, and locality were calculated by multiplying age-cause-state-locality population estimates by the benchmark rates, and the expected deaths were subtracted from observed deaths to yield potentially excess deaths. Age-cause-state-locality potentially excess deaths were summed over age groups to produce cause-state estimates and over states to produce cause-specific estimates for the 10 U.S. Department of Health and Human Services (HHS) public health regions (Figure 1) and the United States. Estimates of potentially excess deaths that were negative, typically from one or two of the benchmark states, were set to zero. For each cause of death, the percentage of deaths that was potentially excess was calculated by dividing the cause-locality–specific estimate of potentially excess deaths by the observed number of deaths for that cause and locality.

The numbers of potentially excess deaths for each cause were assumed to follow a Poisson distribution, and standard errors for both the number and percentage of excess deaths were calculated using standard formulas that incorporated the variance both around the observed and the expected counts (*11*). National trends for nonmetropolitan and metropolitan areas and differences in trends between nonmetropolitan and metropolitan areas in cause-specific age-adjusted death rates during 1999–2014 and in numbers of potentially excess deaths during 2010–2014 were assessed using Joinpoint regression (*12*). Differences in the percentages of deaths that were potentially excess in nonmetropolitan and metropolitan areas were compared using z-tests. Trends and differences discussed in the results were considered statistically significant at p<0.05.

## Results

### **Trends in Annual Age-Adjusted Death Rates, 1999**–**2014**

**FIGURE 2:**
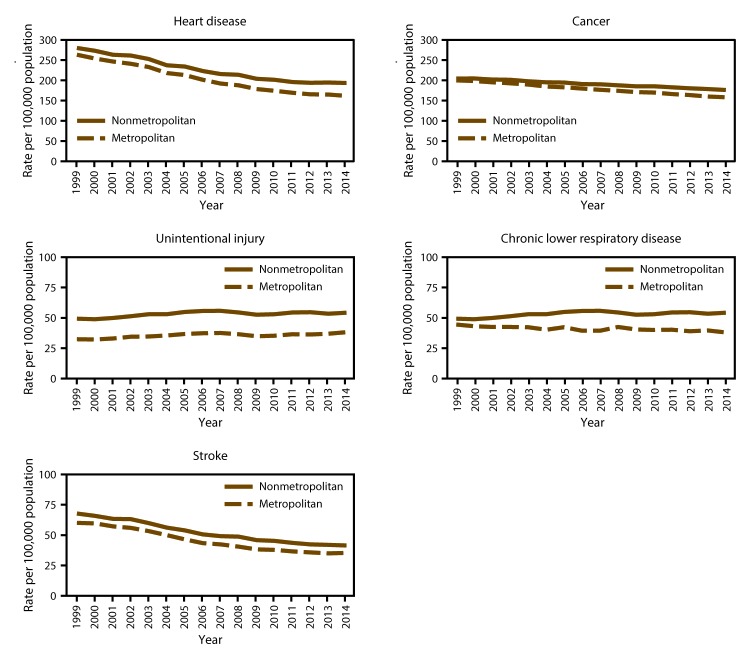
Age-adjusted death rates among persons of all ages for five leading causes of death in nonmetropolitan and metropolitan areas,* by year — National Vital Statistics System, United States, 1999–2014 * Nonmetropolitan and metropolitan areas were identified using the Office of Management and Budget’s 2013 county-based classification scheme. (**Source:** Office of Management and Budget, White House. Revised delineations of metropolitan statistical areas, micropolitan statistical areas, and combined statistical areas, and guidance on uses of the delineations of these areas. Washington, DC: Office of Management and Budget; 2013. https://www.whitehouse.gov/sites/default/files/omb/bulletins/2013/b13-01.pdf)

During 1999–2014, annual age-adjusted death rates for heart disease, stroke, cancer, unintentional injury, and chronic lower respiratory disease were higher in nonmetropolitan areas than in metropolitan areas (Figure 2). Age-adjusted death rates for unintentional injury were approximately 50% higher in nonmetropolitan areas than in metropolitan areas for most of this period. Both in nonmetropolitan and metropolitan areas, annual age-adjusted death rates for heart disease and stroke decreased; however, the rate of decrease for heart disease was slower in nonmetropolitan areas, whereas the rates of decrease for stroke were similar. During 1999–2014, annual age-adjusted death rates for chronic lower respiratory disease decreased in metropolitan areas and increased in nonmetropolitan areas. Although the annual age-adjusted death rates for cancer decreased in both localities during this period, the rate of decrease was slower in nonmetropolitan areas. The annual age-adjusted death rates for unintentional injury increased at similar rates in nonmetropolitan and metropolitan areas. 

### **Potentially Excess Deaths, 2010**–**2014**

**FIGURE 3:**
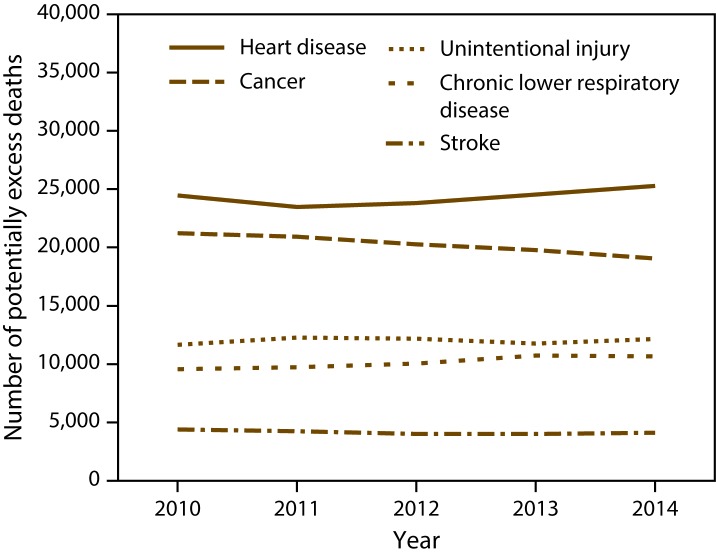
Number of potentially excess deaths* among persons aged <80 years for five leading causes of death in nonmetropolitan areas,† by year — National Vital Statistics System, United States, 2010–2014 * For each age group and cause, the death rates of the three states with the lowest rates during 2008−2010 (benchmark states) were averaged to produce benchmark rates. Potentially excess deaths were defined as deaths among persons aged <80 years in excess of the number that would be expected if the age-specific death rates of the benchmark states occurred across all states. ^†^ Nonmetropolitan areas were identified using the Office of Management and Budget’s 2013 county-based classification scheme. (**Source:** Office of Management and Budget, White House. Revised delineations of metropolitan statistical areas, micropolitan statistical areas, and combined statistical areas, and guidance on uses of the delineations of these areas. Washington, DC: Office of Management and Budget; 2013. https://www.whitehouse.gov/sites/default/files/omb/bulletins/2013/b13-01.pdf)

As the number of persons aged <80 years living in nonmetropolitan areas of the United States decreased from 44.3 million in 2010 to 44.1 million in 2014 (an average of 0.1% per year), the numbers of potentially excess deaths in nonmetropolitan areas decreased for cancer an average of 2.7% per year, increased for chronic lower respiratory disease an average of 3.2% per year, and remained stable for heart disease, stroke, and unintentional injury (Figure 3). In 2014, the number of potentially excess deaths among those aged <80 years for the five leading causes of death in nonmetropolitan areas of the United States were 25,278 from heart disease, 19,055 from cancer, 12,165 from unintentional injury, 10,676 from chronic lower respiratory disease, and 4,108 from stroke.

**FIGURE 4:**
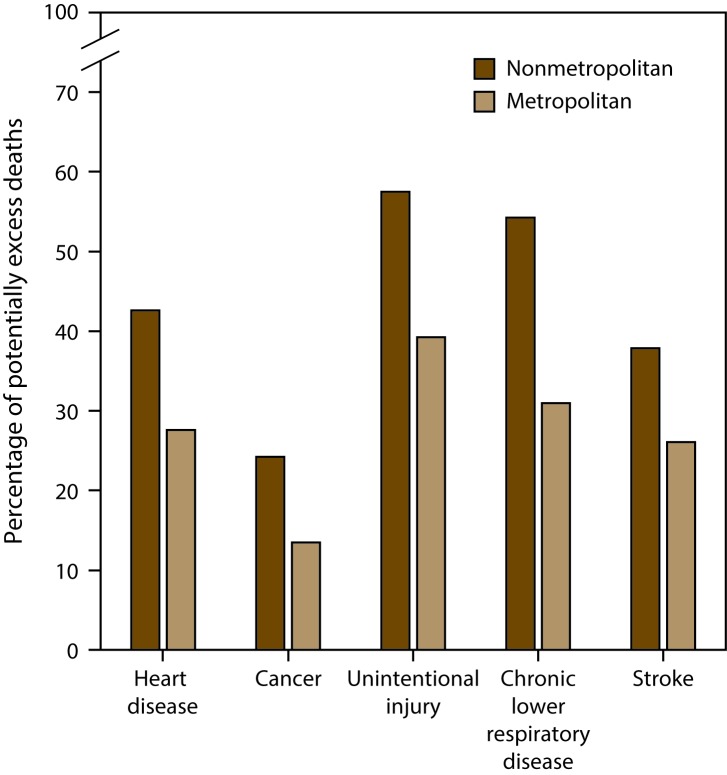
Percentage of potentially excess deaths* among persons aged <80 years for five leading causes of death in nonmetropolitan and metropolitan areas† — National Vital Statistics System, United States, 2014 * For each age group and cause, the death rates of the three states with the lowest rates during 2008−2010 (benchmark states) were averaged to produce benchmark rates. Potentially excess deaths were defined as deaths among persons aged <80 years in excess of the number that would be expected if the age-specific death rates of the benchmark states occurred across all states. ^†^ Nonmetropolitan and metropolitan areas were identified using the Office of Management and Budget’s 2013 county-based classification scheme. (**Source:** Office of Management and Budget, White House. Revised delineations of metropolitan statistical areas, micropolitan statistical areas, and combined statistical areas, and guidance on uses of the delineations of these areas. Washington, DC: Office of Management and Budget; 2013. https://www.whitehouse.gov/sites/default/files/omb/bulletins/2013/b13-01.pdf)

Compared with metropolitan areas, a higher percentage of deaths occurring in nonmetropolitan areas from the five leading causes among those aged <80 years were potentially excess deaths (Figure 4). For example, in 2014, 42.6% of heart disease deaths among persons aged <80 years in nonmetropolitan areas were potentially excess deaths, compared with 27.8% in metropolitan areas. Approximately half of deaths among persons aged <80 years from unintentional injury (57.5%) and chronic lower respiratory disease (54.3%) in nonmetropolitan areas were potentially excess deaths, compared with 39.2% and 30.9%, respectively, in metropolitan areas.

**FIGURE 5:**
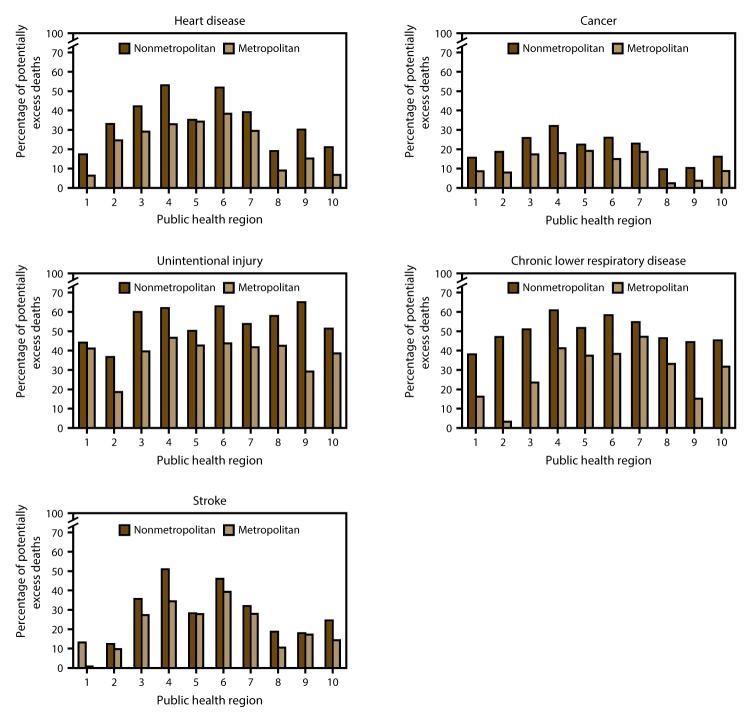
Percentage of potentially excess deaths* among persons aged <80 years for five leading causes of death in nonmetropolitan and metropolitan areas,† by year and public health region^§^ — National Vital Statistics System, United States, 2014 * For each age group and cause, the death rates of the three states with the lowest rates during 2008−2010 (benchmark states) were averaged to produce benchmark rates. Potentially excess deaths were defined as deaths among persons aged <80 years in excess of the number that would be expected if the age-specific death rates of the benchmark states occurred across all states. ^†^ Nonmetropolitan and metropolitan areas were identified using the Office of Management and Budget’s 2013 county-based classification scheme. (**Source:** Office of Management and Budget, White House. Revised delineations of metropolitan statistical areas, micropolitan statistical areas, and combined statistical areas, and guidance on uses of the delineations of these areas. Washington, DC: Office of Management and Budget; 2013. https://www.whitehouse.gov/sites/default/files/omb/bulletins/2013/b13-01.pdf) ^§^ *1* = Connecticut, Maine, Massachusetts, New Hampshire, Rhode Island, and Vermont; 2 = New Jersey and New York; *3* = Delaware, District of Columbia, Maryland, Pennsylvania, Virginia, and West Virginia; *4* = Alabama, Florida, Georgia, Kentucky, Mississippi, North Carolina, South Carolina, and Tennessee; *5* = Illinois, Indiana, Michigan, Minnesota, Ohio, and Wisconsin; *6* = Arkansas, Louisiana, New Mexico, Oklahoma, and Texas; *7* = Iowa, Kansas, Missouri, and Nebraska; *8* = Colorado, Montana, North Dakota, South Dakota, Utah, and Wyoming; *9* = Arizona, California, Hawaii, and Nevada; *10* = Alaska, Idaho, Oregon, and Washington.

The percentages of potentially excess deaths among persons aged <80 years from the five leading causes varied widely across HHS public health regions (Figure 5). In most public health regions, nonmetropolitan areas had a higher percentage of potentially excess deaths among persons aged <80 years than did metropolitan areas. For example, in 2014 in region 9 (Arizona, California, Hawaii, and Nevada), 65.0% of unintentional injury deaths in nonmetropolitan areas were potentially excess deaths, compared with 29.2% in metropolitan areas. Nonmetropolitan areas of region 4 (Alabama, Florida, Georgia, Kentucky, Mississippi, North Carolina, South Carolina, and Tennessee) and region 6 (Arkansas, Louisiana, New Mexico, Oklahoma, and Texas) had the highest percentages of heart disease, cancer, chronic lower respiratory disease, and stroke deaths that were potentially excess deaths. Metropolitan areas of region 1 (Connecticut, Maine, Massachusetts, New Hampshire, Rhode Island, and Vermont) had the lowest percentages of heart disease and stroke deaths that were potentially excess deaths, and metropolitan areas of region 2 (New Jersey and New York) had the lowest percentages of unintentional injury and chronic lower respiratory disease deaths that were potentially excess deaths.

## Discussion

During 1999–2014, nonmetropolitan areas had higher age-adjusted death rates from the five leading causes of death than metropolitan areas, consistent with previous reports (*13*). During this period, the age-adjusted death rates for heart disease, cancer, chronic lower respiratory disease, and stroke in metropolitan areas decreased. In nonmetropolitan areas, the rates for heart disease, cancer, and stroke decreased but increased for chronic lower respiratory disease. Both in nonmetropolitan and metropolitan areas, the rates for unintentional injury increased. During this period, differences in age-adjusted death rates between nonmetropolitan and metropolitan areas either persisted or increased for the five leading causes of death. Consistent with previous studies, considerable numbers of deaths among persons aged <80 years from the five leading causes of death were identified as potentially excess deaths nationally (*7*,*8*). Data from this report indicate that the percentage of deaths among persons aged <80 years that are potentially excess deaths was higher in nonmetropolitan areas than in metropolitan areas, which is a new finding. Moreover, this difference was observed in most public health regions, even as percentages varied widely across the United States.

Previous research has shown that residents of nonmetropolitan areas, compared with residents of metropolitan areas, report poorer physical and mental health and have higher rates of health risk factors for the leading causes of death, including factors such as cigarette smoking, obesity, physical inactivity during leisure time, and not wearing car seat belts (*2*,*3*,*14*). They tend to have less access to health care services and to be less likely to receive preventive services (*5*). In addition, they are more likely to be uninsured or underinsured, delay seeking care, live in poverty, and have lower educational attainment (*1*,*5*). The high percentage of potentially excess deaths reported in nonmetropolitan areas might be related to the poorer health, limited socioeconomic resources, restricted access to high-quality emergency and specialty care such as trauma centers and stroke centers, and reduced emergency medical service capability (*15*) that exist in many nonmetropolitan communities. Although a greater number of potentially excess deaths occur in metropolitan areas because of the larger populations, these findings suggest that a greater percentage of deaths in nonmetropolitan areas might be potentially excess deaths and thus are relevant for public health prevention efforts focused on rural populations. Ongoing work by CDC to reduce rates of smoking (*16*) and obesity (*17*) are particularly important because these risk factors contribute to increased risk for heart disease, stroke, chronic lower respiratory disease, and cancer (*18*,*19*). In addition, CDC’s efforts to increase access to preventive services, such as cancer screening (*20*) and cardiovascular risk reduction (*21*), address barriers that persons living in nonmetropolitan areas might face.

## Limitations

The findings in this report are subject to at least five limitations. First, age-adjusted rates are index measures that do not represent actual deaths but are appropriate for comparisons across populations with different age distributions. Second, standard errors of estimates of potentially excess deaths were calculated using standard formulas that incorporate the variance around both the observed and the expected counts. Less conservative methods for calculating standard errors might identify additional differences in potentially excess deaths between nonmetropolitan and metropolitan areas that do not achieve statistical significance using the more conservative approach reported here. Third, the same benchmarks based on the three states with the lowest death rates were applied to nonmetropolitan and metropolitan areas. Although nonmetropolitan areas might have characteristics that make deaths harder to prevent, such as long travel distances and fewer community resources to support health, numbers of potentially excess deaths both in nonmetropolitan and metropolitan areas can likely be reduced through public health and health care programs. Fourth, estimates of potentially excess deaths using historical benchmarks (e.g., 2008–2010) might not reflect the progress that could be made in a later year. For example, the estimates of potentially excess deaths for cancer based on 2010 benchmarks are lower than those based on 2014 benchmarks because of decreases in cancer deaths during 2010–2014. Finally, the county-based metropolitan-nonmetropolitan classification scheme does not account for variation in population density within counties. For example, large metropolitan counties might include areas that are defined as rural by the U.S. Census Bureau. In addition, assessing potentially excess deaths using the nonmetropolitan and metropolitan classifications masks important differences within urban and rural areas. For example, considerable variation occurs in settlement patterns and density among nonmetropolitan counties; some include small towns, and others are classified as frontier counties with no urban areas. Considerable variation in measures of health occurs among metropolitan counties; suburban metropolitan counties tend to have substantially better health outcomes than inner-city metropolitan counties (*2*,*3*).

## Future Directions

This report demonstrates the value of disaggregating nonmetropolitan and metropolitan area deaths and potentially excess deaths. Trends in death rates and potentially excess deaths over a longer period might provide a better understanding of factors that contribute to differences. Examination of county-level patterns within states (and other jurisdictions) might be used to help determine where to allocate resources and assistance in rural areas with larger numbers of potentially excess deaths. Comparison with tools such as the CDC Interactive Atlas of Heart Disease and Stroke (http://www.cdc.gov/dhdsp/maps/atlas/index.htm) might help identify the social determinants, health care infrastructure, and public policies that attenuate or exacerbate mortality in specific nonmetropolitan areas. Detailed community-based case-control studies comparing areas with the highest and lowest death rates might clarify how various risk factors and community-wide social determinants of health affect mortality in rural and urban areas. In addition, consideration of other methods for developing benchmark rates might be helpful. Other methods include benchmarks based on the nonmetropolitan areas with the lowest death rates, alternative approaches for accounting for uncertainty around estimates of potentially excess deaths such as Monte Carlo procedures or bootstrapping, potentially excess deaths among persons aged >80 years, and potentially excess deaths from other causes, especially causes that are more prevalent in rural areas.

## Conclusion

Nonmetropolitan areas have higher age-adjusted death rates and greater percentages of potentially excess deaths from the five leading causes of death. Routine tracking of potentially excess deaths from the five leading causes of death in nonmetropolitan and metropolitan areas might help public health officials monitor important rural health disparities and select effective programs and policies to improve the health of residents of rural areas. Additional information on potentially excess deaths might be used to evaluate the success of public health interventions and to help determine where to allocate resources in areas with the greatest need. State and local public health officials in rural areas might seek advice from officials in rural areas with fewer potentially excess deaths for ways to reduce mortality in their jurisdictions or increase coordination with urban areas to ensure rural residents have timely access to specialized services. More detailed data on age-adjusted mortality and potentially excess deaths are available as supplemental materials (https://stacks.cdc.gov/view/cdc/43149 and https://stacks.cdc.gov/view/cdc/43148).
